# ECMO Predictors of Mortality: A 10-Year Referral Centre Experience

**DOI:** 10.3390/jcm11051224

**Published:** 2022-02-24

**Authors:** Benedikt Treml, Robert Breitkopf, Zoran Bukumirić, Mirjam Bachler, Johannes Boesch, Sasa Rajsic

**Affiliations:** 1 General and Surgical Intensive Care Unit, Department of Anaesthesiology and Critical Care Medicine, Medical University Innsbruck, 6020 Innsbruck, Austria; benedikt.treml@tirol-kliniken.at (B.T.); mirjam.bachler@tirol-kliniken.at (M.B.); johannes.boesch@tirol-kliniken.at (J.B.); 2 Transplant Surgical Intensive Care Unit, Department of Anaesthesiology and Critical Care Medicine, Medical University Innsbruck, 6020 Innsbruck, Austria; robert.breitkopf@tirol-kliniken.at; 3 Institute of Medical Statistics and Informatics, Faculty of Medicine, University of Belgrade, 11000 Belgrade, Serbia; zoran.bukumiric@med.bg.ac.rs

**Keywords:** adverse events, cardiogenic shock, complications, extracorporeal life support, ECMO, hypothermia, mortality, respiratory failure, rewarming, risk factors

## Abstract

Background: Extracorporeal membrane oxygenation (ECMO) is a specialised life support modality for patients with refractory cardiac or respiratory failure. Multiple studies strived to evaluate the benefits of ECMO support, but its efficacy remains controversial with still inconsistent and sparse information. Methods: This retrospective analysis included patients with ECMO support, admitted between January 2010 and December 2019 at a tertiary university ECMO referral centre in Austria. The primary endpoint of the study was overall all-cause three-month mortality with risk factors and predictors of mortality. Secondary endpoints covered the analysis of demographic and clinical characteristics of patients needing ECMO, including incidence and type of adverse events during support. Results: In total, 358 patients fulfilled inclusion criteria and received ECMO support due to cardiogenic shock (258, 72%), respiratory failure (88, 25%) or hypothermia (12, 3%). In total, 41% (145) of patients died within the first three months, with the median time to death of 9 (1–87) days. The multivariate analysis identified hypothermia (HR 3.8, *p* < 0.001), the Simplified Acute Physiology Score III (HR 1.0, *p* < 0.001), ECMO initiation on weekends (HR 1.6, *p* = 0.016) and haemorrhage during ECMO support (HR 1.7, *p* = 0.001) as factors with higher risk for mortality. Finally, the most frequent adverse event was haemorrhage (160, 45%) followed by thrombosis. Conclusions: ECMO is an invasive advanced support system with a high risk of complications. Nevertheless, well-selected patients can be successfully rescued from life-threatening conditions by prolonging the therapeutic window to either solve the underlying problem or install a long-term assist device. Hypothermia, disease severity, initiation on weekends and haemorrhage during ECMO support increase the risk for mortality. In the case of decision making in a setting of limited (ICU) resources, the reported risk factors for mortality may be contemplable, especially when judging a possible ECMO support termination.

## 1. Introduction

Extracorporeal membrane oxygenation (ECMO) is a specialised, advanced life support modality for critically ill patients with refractory cardiac or respiratory failure. The first report of prolonged extracorporeal oxygenation and perfusion of a patient suffering from severe acute respiratory distress syndrome (ARDS) dates from 1971 [1] and is often seen as the beginning of ECMO support as we know it today [2]. Although extracorporeal membrane oxygenation is often referred to as a therapy, it is actually a temporary support for cardiorespiratory failure, bridging time for recovery or permanent assist. It is used as a last resort in severe cardiogenic shocks as venoarterial (va-ECMO) and in respiratory failures as a venovenous (vv-ECMO) configuration [3]. Furthermore, ECMO could be used in a variety of clinical presentations, including the resuscitation of patients with severe traumas, extracorporeal-assisted rewarming (ECAR) of accidental deep hypothermia and bridge to lung or heart transplant. In the last decade, the use of ECMO support in cases of an out-of-hospital cardiac arrest through prehospital emergency medical service has been described by some facilities [4,5]. The Extracorporeal Life Support Organization (ELSO) recommends the consideration of ECMO support in cases of cardiorespiratory failure with a high risk of mortality (50%), and initiation in most circumstances when the mortality risk reaches 80%, in selected patients [6,7].

The outbreak of coronavirus disease 2019 (COVID-19) overwhelmed the existing intensive care unit (ICU) capacities, as most patients needed prolonged mechanical ventilation and diverse extracorporeal organ support. Acute respiratory distress syndrome, a rapidly progressive inflammatory syndrome impairing oxygenation, can be a consequence of a COVID-19 progression and a possible indication for ECMO use [8]. A recent meta-analysis of 22 COVID-19 ECMO studies reported 37% in-hospital mortality during the first year of the pandemic. These results are consistent with the non-COVID-19-related ARDS mortality [9].

According to the ELSO registry, based on the data from 521 centres, more than 160,000 ECMOs were employed until the end of 2020. The number of ECMO support cases increased gradually in the last ten years, from 3446 in 2010 to more than 18,000 in 2020. Moreover, the number of ECMO centres tripled in that period [10]. The reported survival rate of all adult ECMO patients was 61%, with a 49% survival further to discharge or transfer [10].

However, the benefit of this potentially life-saving support is still the subject of discussion. Despite the ongoing progress of modern intensive care medicine, severe ARDS is still associated with high mortality [11], as it can be challenging to achieve a sufficient pulmonary gas exchange under the limitations of a lung-protective ventilation regime. Hence, selected patients may benefit from vv-ECMO by avoiding an additional ventilator-associated lung injury (VALI) [12,13]. In the cases of cardiogenic shock and va-ECMO use, the available information on patient benefits is likewise scarce and a subject of debate [14,15,16]. Multiple studies strived to evaluate the potential benefit of ECMO support, but due to methodological issues, its efficacy remains controversial [17,18,19,20].

Therefore, the aim of this study is to investigate the overall all-cause three-month mortality and to identify possible predictors and risk factors for mortality. Moreover, this study provides a summary and comparison of the demographic and clinical characteristics of a mixed population of critically ill patients undergoing ECMO support while focusing on adverse events and outcomes.

## 2. Materials and Methods

### 2.1. Study Population

We retrospectively reviewed the electronic medical charts of all patients admitted to the two intensive care units between January 2010 and December 2019. These tertiary ICUs of the department for anaesthesiology and critical care medicine of the Medical University Innsbruck, Austria treat medical, surgical and trauma patients. All consecutive critically ill patients undergoing ECMO support were enrolled. Excluded were patients younger than 10 years, with an ECMO support duration less than six hours, multiple ECMO initiations or with incomplete data sets.

### 2.2. ECMO Support and Anticoagulation

The ECMO programme is perpetually available in our centre. The decision on the ECMO support initiation is made by the joint judgement of a cardiac surgeon, a cardiac anaesthesiologist and an intensivist on call. The institutional standard operating procedure protocol for ECMO initiation regulates possible indications, contraindications and the ECMO initiation report form. The ECMO system consisted of a centrifugal pump, a hollow-fibre oxygenator, UFH-coated circuit, arterial and venous cannulas and an integrated heat exchanger for temperature regulation.

ECMO cannulation is usually established through percutaneous femoral or jugular access, with distal leg perfusion in va-ECMO configuration. In the case of an unsuccessful percutaneous insertion, direct cannulation with incision was performed. Anticoagulation of ECMO patients was conducted according to the standard operating procedure protocol and based on the ELSO Anticoagulation Guideline [21]. Unfractionated heparin was used as the first choice for anticoagulation, initially with 5–20 IU/kg/hour and targeted aPTT of 50–70 s. In the case of inadequate anticoagulation or suspected heparin-induced thrombocytopenia type 2, argatroban was used. Monitoring and adaptation of anticoagulation were performed according to the activated clotting time (ACT), aPTT, CT INTEM in the ROTEM^®^, antifactor Xa assay activity or blood drug concentration.

The ECMO weaning protocol included stepwise reduction of extracorporeal blood flow or sweep gas after successful treatment of the initial cause. Potential recovery of the heart was regularly evaluated using transesophageal echocardiography, while recovery of pulmonary function was monitored through improvement of respiratory coefficient, ventilation parameters and radiological imaging. After clinical judgement and with ECMO support less than 30% of the total, a trial off was initiated. Discontinuation of ECMO and decannulation were conducted according to ELSO recommendations [7]. In the case of futility (due to severe brain damage, irreversible heart or lung damage, or multiple organ failure), ECMO support was terminated promptly. In certain cases, organ explanation and donation were considered.

### 2.3. Data Acquisition

We obtained sociodemographic data of the patients; data on disease severity prior to ECMO initiation, underlying disease and indication for ECMO support, type of ECMO support, cardiopulmonary resuscitation before or during ECMO implantation, ECMO support duration; information on adverse events (including type, date and location), date and cause of death; use of anticoagulation, transfusion of blood and coagulation products; laboratory parameters with coagulation status including platelets count (g/L), fibrinogen (modified Clauss method, mg/dL), rotational thromboelastometry (ROTEM), factor XIII (%), activated partial thromboplastin time (seconds), prothrombin time (%), international normalised ratio and antithrombin (%); other laboratory parameters as erythrocytes (T/L), haematocrit (l/L), haemoglobin (g/L), white blood cells count (g/L), C-reactive protein (mg/dL) and procalcitonin (µg/L); and finally data on mortality in different periods.

Laboratory data were recorded starting within 24 h before ECMO initiation, and daily during the whole support period, and continued on the third and tenth day after ECMO termination. The observation period of laboratory parameters was limited to 14 days.

Two authors independently checked each medical chart and extracted the data in the predesigned case report form.

### 2.4. Outcomes

The primary endpoint of the study was overall all-cause three-month mortality with risk factors and predictors of mortality. Secondary endpoints included the comparison (ECMO survivors versus nonsurvivors) of demographic and clinical characteristics of patients needing ECMO support, including incidence and type of adverse events during support. Finally, we report on subgroup analyses based on the ECMO type and indications.

Patient and clinical characteristics were collected as described in Section 2.3. Reported adverse events comprised bleeding, coagulopathy, thromboembolic events and sepsis. Bleeding was observed for a period of 14 days, and any other bleeding event thereafter was considered to be unrelated to the ECMO. Bleeding events were defined as minor or major, according to the ELSO definition [21]. A major bleeding event was defined as clinically overt bleeding associated with a haemoglobin decrease of at least 2 g/dL over 24 h or administration of two or more red blood concentrate units over the same period. Any pulmonary, retroperitoneal, bleeding involving the central nervous system or requiring surgical intervention was also considered as a major event. A minor bleeding event was defined as any other noticeable bleeding [21]. In the case of multiple sources or bleeding events, only the date of the first bleeding event was recorded.

Information on thromboembolic events (localisation, date of identification and type of event) was collected from the electronic medical documentation and radiological reports, covering the whole ECMO support period and additional two weeks after ECMO termination. The retrospective confirmation of thromboembolism was only possible if the radiological investigation was performed. Thromboembolic events included central arterial and venous (heart, aorta and pulmonary artery) or peripheral thrombus formation (any peripheral artery or vein), embolisation (i.e., ischaemic stroke, extremities, etc.), central vascular catheters or ECMO cannula and any mixed arterial and venous thrombosis.

The cause of death was collected from electronic medical charts or post-mortem examination reports. Based on the recorded date of death, mortality in different periods was calculated. The subgroup analyses included evaluation of patient and clinical characteristics based on the ECMO indication, type and initiation day.

This retrospective study was approved by the Ethics Committee of the Medical University of Innsbruck, Austria (Ethics Committee Number: 1274/2019).

### 2.5. Statistical Analyses

A statistician not involved in patient assessment or study procedures performed statistical analyses using SPSS (Version 22.0. Released 2013, Armonk, NY, USA: IBM Corp.) and the R program (version 4.0.2; free software for statistical computing and graphics—R Core Team 2020: a language and environment for statistical computing; R Foundation for Statistical Computing, Vienna, Austria). All statistical assessments were two-sided, and a significance level of 0.05 was applied. Missing data were not analysed. Depending on the normality of the distribution and the type of variables, results are presented as frequency (percent), mean with standard deviation and median (range, minimum–maximum). For parametric data, independent samples *t*-test or ANOVA were used, and Mann–Whitney U or Kruskal–Wallis test for ordinal and numeric data with non-normal distribution. Chi-square test and Fisher’s exact test were used to test differences between nominal data (frequencies). In the univariate Cox regression analyses, the effect of each potential risk factor on mortality was estimated, and all significant covariates were further assessed in the multivariate model. The significance level for the multivariate model was set to 0.1. To estimate the survival function in dependence of ECMO support indication (respiratory failure, cardiogenic shock or hypothermia) the log-rank test was used. To determine the cut-off values of Simplified Acute Physiology Score III (SAPS III score) for predicting mortality, the receiver operating characteristics (ROC) analysis was used.

## 3. Results

### 3.1. Patient and ECMO Characteristics

Over a period of ten years, 568 patients received ECMO support. After screening all medical charts, 392 patients met inclusion criteria, with 358 patients showing complete data sets (Table 1 and Table 2). Cardiogenic shock (117, 33%), heart valve disease (57, 16%) and ARDS (50, 14%) were the main ICU admission reasons (Supplementary Figure S1). The median SAPS III score was 67 (28–117), and 18% (65) of patients were mechanically resuscitated. The median length of ICU stay was 18 (1–170) days.

The va-ECMO configuration was used in 284 (79%) patients (Table 2). Extracorporeal membrane oxygenation was usually commenced on working days and immediately before ICU admission, with a median overall duration of six days. Anticoagulation was realised with heparin (278, 78%), argatroban (32, 9%) and epoprostenol. Due to coagulopathy and/or life-threatening bleeding, 41 (12%) patients did not receive any anticoagulation. Finally, 277 (77%) patients survived beyond ECMO support and 216 (60%) were discharged alive from the hospital.

### 3.2. Outcomes

**Figure 1:**
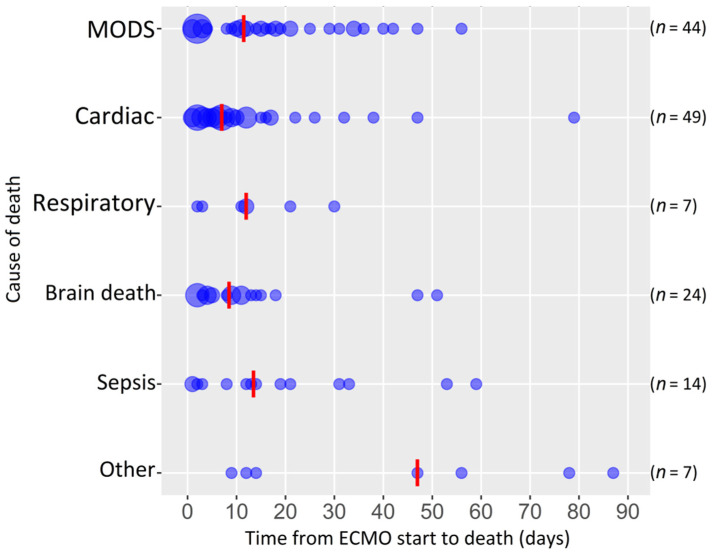
Causes of death within three months from ECMO initiation (circle size depends on the number of patients; red line presents the median; *n* = 145). Abbreviations: MODS, multiple organ dysfunction syndrome; ECMO, extracorporeal membrane oxygenation.

**Figure 2:**
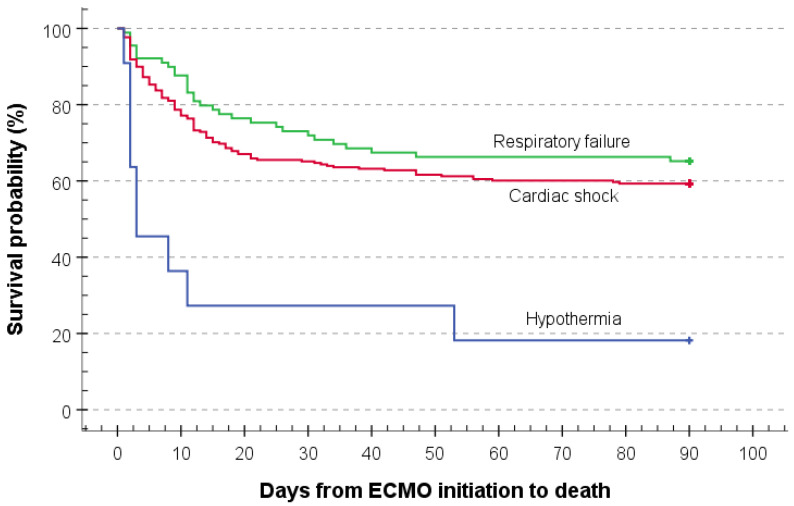
Kaplan–Meier mean estimate of all-cause three-month mortality in regard to ECMO indication (*n* = 358) was 65 days for respiratory failure (*n* = 88; 95% CI: 57.5–72.4), 59 days for cardiogenic shock (*n* = 258; 95% CI: 54.4–63.9) and 24 days for hypothermia (*n* = 12; 95% CI: 3.9–44.3). Abbreviations: ECMO, extracorporeal membrane oxygenation; CI: confidence intervals.

Overall three-month all-cause mortality was 41% (145 patients, Table 1), and the main causes of death were cardiac failure, multiple organ dysfunction syndrome and brain death (Figure 1). In total, 23% (81) of patients died during ECMO support and 37% (131) during the whole ICU stay (Table 1). The median time from ECMO support initiation to death was 9 (1–87) days (Table 1). The Kaplan–Meier estimate mean of all-cause three-month mortality was 59.5 days (95% CI: 55.5–63.5, Supplementary Figure S2). The Kaplan–Meier estimate in regard to ECMO indication is presented in Figure 2.

**Figure 3:**
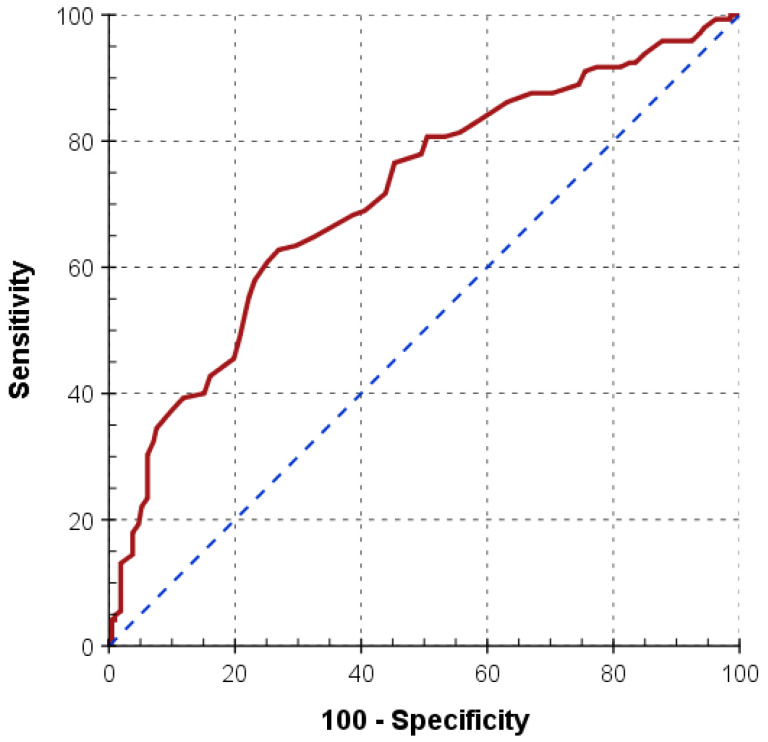
Receiver operating characteristic curve (ROC) with the cut-off SAPS III score for predicting mortality of more than 69 points (*n* = 357; AUC = 0.712, *p* < 0.001; sensitivity = 62.8, specificity = 73.1). Abbreviations: SAPS III, Simplified Acute Physiology Score III; AUC, area under the curve.

Within 24 h prior to ECMO initiation, nonsurvivors had higher C-reactive protein levels and shorter InTEM clotting times, the latter ranging within normal limits (Supplementary Table S1). Deceased patients had significantly higher Simplified Acute Physiology Score III (SAPS III) and Sequential Organ Failure Assessment (SOFA) scores at admission, were more often resuscitated and had a shorter length of ICU stay (Table 1). The ROC analysis determined a SAPS III score of 69 points (AUC = 0.71, *p* < 0.001; sensitivity 62.8, specificity 73.1) being the optimal cut-off for predicting mortality (Figure 3). Finally, nonsurvivors had more often coagulopathy and serious bleeding, consequently needing more substitution of blood products. Sepsis was diagnosed more often in this group (Table 3).

The most frequent adverse event during ECMO support was haemorrhage (160, 45%) followed by thrombosis (82, 23%; Table 3). Haemorrhage appeared in 86 (24%) patients already during the first ECMO day, and in 117 (33%) within the first three days. Moreover, 56% (81) of nonsurvivors experienced any and 37% (53) major haemorrhage, being significantly higher when compared to survivors (Table 3).

The univariate Cox regression analysis identified a higher SAPS III and SOFA score, hypothermia as indication, ECMO initiation on weekends and resuscitation before or during ECMO initiation as independent predictors of three-month mortality. Moreover, haemorrhage and sepsis presented adverse events with an increased risk for mortality (Supplementary Table S6). Finally, a higher SAPS III score, hypothermia as indication, ECMO initiation on weekends and haemorrhage had increased hazard ratio for three-month mortality in the multivariate Cox regression model (Table 4).

### 3.3. Subgroup Analyses

Patients who received extracorporeal support on weekends had higher mortality after three months and higher SAPS III scores in the subgroup analysis (Supplementary Table S2). The majority of these patients had nonsurgical cardiac failure, were slightly younger and experienced haemorrhage less often.

Based on the ECMO support indications, patients with accidental hypothermia had the highest mortality (Supplementary Table S3 and Figure 2). These patients were younger, with lower body mass index and higher SAPS III score. Five patients have been resuscitated before ECMO initiation, and ten died during the ICU stay. Cardiac failure was the main cause of death in patients with cardiac shock and multiple organ failure syndrome in the respiratory failure group. Brain death (7/10 patients) dominated in the group of patients with accidental hypothermia. (Supplementary Table S3).

There was no difference in haemorrhage and thromboembolic events between the groups in regard to ECMO support indication. Sepsis occurred more frequently in patients with respiratory failure (37, 42%). Moreover, patients with respiratory failure were younger, seldom resuscitated, had longer ECMO duration and ICU stay when compared to patients with cardiac shock.

The analysis of patients receiving va-ECMO showed similar findings such as the main analysis (Supplementary Table S4). On the contrary, nonsurvivors receiving vv-ECMO had a significantly higher SAPS III score, were seldom resuscitated, had a longer period from ICU admission to ECMO initiation and needed more often substitution from packed red blood cells and platelets (Supplementary Table S5). The main adverse event in the vv-ECMO group was sepsis (35, 47%).

## 4. Discussion

In this retrospective study from a Central European tertiary university centre, we report on overall all-cause three-month mortality, demographic and clinical characteristics of patients undergoing ECMO support due to cardiogenic shock, respiratory failure or hypothermia. We summarise that more than half of the ECMO patients can successfully be supported during life-threatening conditions and finally rescued by extracorporeal life support. We identify hypothermia as the most unfavourable indication in regard to patient outcome. Higher SAPS III and SOFA scores, haemorrhage during ECMO support and ECMO initiation on weekends are associated with higher three-month mortality. Finally, the most favourable outcome has been seen in patients with respiratory failure, which is in line with current data [22], the vast majority of patients could successfully be weaned from the ECMO support.

Technological development has led to increased use of ECMO support regardless of increasing comorbidities. There is an ongoing contemporary trend to treat sicker patients, either as an option to improve quality of life or as a last life-saving measure. This resulted in the development and popularisation of diverse (extracorporeal) organ supports and replacement therapies, but also in an increase in ICU mortality. Gray et al. reported on experience from an ECMO referral centre in the USA, observing a decreased survival rate in the last 12 years of a 38-year observation period [23]. However, the study population comprised mostly neonates and paediatric patients (73%). In the subgroup analysis of adult patients only, a minimal increase in survival is reported [23]. In our work, we did not identify any difference in mortality during the observation period, most probably due to shorter study duration and the exclusion of paediatric patients. Moreover, the vast majority of patients were admitted due to cardiogenic shock, heart valve disease or ARDS (Supplementary Figure S1). Finally, it is to be expected that the continuous advancement of new ECMO techniques, anticoagulation management, critical care development and complications management will lead to increased survival over time. Due to the expansion of ECMO support indications, this salvage option is increasingly offered to extremely sick patients, accruing the potential of poor outcome.

Based on the mortality risk, ELSO published a general list of ECMO indications but still left scope for individualised risk–benefit considerations [7]. However, most contraindications are only relative, including nonrecoverable comorbidity, recent or expanding CNS haemorrhage, risk of systemic bleeding with anticoagulation, terminal malignancy or age. There is no specific age cut-off, but an age-related risk should be considered according to ELSO guidelines [7,24]. The newest COVID-19 consensus document from ELSO consequently defines the age of 65 years as a relative and advanced age as an absolute contraindication for ECMO initiation [25].

We did not find any association of age and mortality in our cohort of critically ill adults, with the oldest patient being 87 and the oldest survivor 85 years. Moreover, we initiated ECMO support in 187 patients older than 60 years (92 older than 70 years) with 54% three-month and 52% one-year survival. This could be partly explained by a very fit elderly population being physically active, especially in the mountainous Western part of Austria. Given the Austrian life expectancy of 82 years at birth in 2019 [26], and based on our own clinical experience, advanced age alone should not be a contraindication for the ECMO support initiation. The severity of disease in combination with comorbidities, frailty and rehabilitation potential should constitute the base of decision making. Moreover, based on the recommendations of the Austrian Society of Anaesthesiology, Resuscitation and Intensive Care Medicine and a recent study from Spain, in case of limited resources age alone should not be taken as a triage factor [27,28].

The use and accuracy of prognostic scores in critical care is still a subject of debate. We perceived both higher SAPS III and SOFA scores as independent predictors for three-month mortality. Consistent with the literature, patients not surviving ECMO support had a SAPS III score of 73 points, which was 11 points higher than in survivors [29,30]. For the first time, we report on an optimal cut-off value of the SAPS III score for prediction of mortality. We found a SAPS III score exceeding 69 points to bear an increased risk of three-month mortality for any ECMO indication. The literature on predicting scores for ECMO patients is sparse and heterogeneous, limiting comparison with other studies. The majority of publications did not report on the SAPS III score, and only a few reported on the Acute Physiology and Chronic Health Evaluation score (APACHE) as being associated with mortality [31,32].

We identified hypothermia, ECMO initiation on weekends and haemorrhage as factors increasing the risk for mortality. Hypothermic cardiac arrest is an important indication for extracorporeal circulatory support, with still high mortality due to most often underlying primary asphyxia. In our study population, patients with hypothermia had a more than threefold higher chance of death, with only 2 out of 12 patients surviving (17%). Both survivors, initially found with a core temperature of 24 °C, after cold-water immersion and a fall in a crevasse, had a great neurological outcome. The majority of our hypothermic patients died due to asphyxia-related brain death within the first 3 days. A systematic review and meta-analysis including 464 patients reported a nearly double overall survival rate (37%) compared to our results [33]. This discrepancy could be due to the aetiology of hypothermia, as the mentioned review analysed a mixed population from 23 studies, whereas asphyxia-induced cardiac arrests (after avalanche burial and immersion) dominated in our work.

Interestingly, patients with ECMO initiation during weekends had increased mortality compared to other patients. This “weekend effect” is extensively described in the literature for both elective and emergency surgical or nonsurgical patients, with most probably a heterogeneous basis [34,35]. In our work, these patients had a higher SAPS III score only. This may be explained by the fact that on weekends emergency procedures dominate over routine work, which increases the SAPS III score by five points.

In the subgroup analysis of va-ECMO and vv-ECMO, we found that nonsurvivors had higher disease severity scores in both ECMO configurations. Furthermore, deceased vv-ECMO patients spent more time at ICU before the ECMO was initiated, being in line with the literature. Patients with va-ECMO experienced bleeding events commonly on the first ECMO day, while those receiving vv-ECMO on the fourth day. Moreover, patients needing vv-ECMO support had 47% sepsis, and within all analysed groups, sepsis was more often in deceased patients.

Finally, the multivariate analysis identified haemorrhage as a major risk factor for mortality with an almost two times higher chance of death. Haemorrhage is one of the most common and serious complications during ECMO support, with the potential for permanent injury or death [36]. In our cohort, 45% of patients experienced haemorrhage, with the vast majority within the first three days. Of the total 145 nonsurvivors, 81 (56%) experienced any and 53 (37%) major haemorrhage, being significantly higher when compared to survivors. In the mixed literature from va-ECMO and vv-ECMO configurations, haemorrhage is found to be directly associated with poor clinical outcome [36,37]. Due to various patient factors, ECMO indications, interactions of the artificial surface of the ECMO circuit and individualised anticoagulation protocols, the identification of risk factors for haemorrhage in the population of critically ill patients is at least complex, and further research is warranted.

Both a higher SOFA score and sepsis have been shown to increase the hazard ratio for mortality, being poor prognostic markers for patients with ECMO support. ECMO could be used as a salvage option for severe sepsis-induced cardiogenic shock, even if characterised with very high mortality [32,38]. However, early clinical suspicion, provision of blood cultures and timely initiation of empirical antimicrobial therapy can substantially decrease the mortality of sepsis [38,39].

This study has several limitations. Due to its retrospective nature, a selection bias cannot be excluded. Although this is one of the largest European retrospective studies with the consecutive inclusion of patients, we cannot rule out the potential effect of missing variables. Furthermore, it is complex to discriminate ECMO-related adverse events from potential complications of an underlying disease. However, reported major bleeding events and thrombosis are most probably a consequence of ECMO support and distorted coagulation. We used the recently published ELSO bleeding definition to classify bleeding [21], but some complications may have been overlooked or missed if no additional diagnostic procedure was performed. Moreover, the retrospective confirmation of thrombosis was only possible if the radiological investigation was performed, which could lead to underestimation of thrombosis incidence in our study. However, due to the liberal approach to diverse diagnostic modalities, this chance may be rather small. Lastly, our study comprises a large cohort of patients in comparison to literature, but larger samples and further studies are needed to elucidate risk factors for reported clinical and patient outcomes and potential mortality predictors during and after ECMO support.

## 5. Conclusions

In this retrospective study from an ECMO referral centre, 41% (145) of patients died within the first three months. We identified higher SAPS III score, hypothermia and ECMO initiation on weekends as significant predictors of mortality, in contrast to the often-speculated risk factors age and cardiopulmonary resuscitation. Haemorrhage is the main adverse event during ECMO support associated with a worse outcome. Finally, ECMO-support-related decision making could be additionally supported by comprehension of the reported risk factors for mortality. This is especially true when judging rather a possible ECMO termination than commencement in light of the already-stretched (ICU) resources.

## Figures and Tables

**Table 1:** Baseline demographic and clinical characteristics of included patients (*n* = 358, all-cause three-month mortality).

Patient Characteristics *	All Patients (*n* = 358)	Survivors (*n* = 213)	Nonsurvivors (*n* = 145)	*p*-Value	Missing Data (*n*/Total)
Age (years)	58.6 ± 15.9	57.6 ± 15.9	60.2 ± 15.9	0.135	0/358
<30 31–45 46–60 61–70 >71	25 (7.0) 40 (11.2) 106 (29.6) 95 (26.5) 92 (25.7)	17 (8.0) 24 (11.3) 71 (33.3) 50 (23.5) 51 (23.9)	8 (5.5) 16 (11.0) 35 (24.1) 45 (31.0) 41 (28.3)	0.224	0/358
Male sex	251 (70.1)	151 (70.9)	100 (69.0)	0.725	0/358
Height (cm)	172 ± 10.0	173 ± 9.2	171 ± 11.1	0.133	10/358
Weight (kg)	80.8 ± 17.7	81.1 ± 17.6	80.4 ± 17.8	0.731	8/358
Body mass index (kg/m^2^)	27.2 ± 5.2	27.0 ± 5.1	27.5 ± 5.4	0.448	10/358
SAPS III score	67 (28–117)	62 (28–112)	73 (31–117)	<0.001	1/358
SAPS III score predicted mortality (%)	50 (1–96)	40 (1–95)	62 (2–96)	<0.001	1/358
SOFA score	12 (2–21)	11 (3–21)	12 (2–21)	0.003	0/358
SOFA respiratory	2 (0–4)	2 (0–4)	2 (0–4)	0.451	
SOFA coagulation	1 (0–4)	1 (0–4)	1 (0–4)	0.722	
SOFA liver	1 (0–4)	1 (1–3)	0 (0–4)	0.383	
SOFA cardiovascular	4 (0–4), mean 3.5	4 (0–4), mean 3.4	4 (0–4), mean 3.7	<0.001	
SOFA neurology	4 (0–4), mean 2.9	4 (0–4), mean 2.7	4 (0–4), mean 3.2	0.002	
SOFA renal	1 (0–4)	1 (0–4)	1 (0–4)	0.070	
CPR before ECMO initiation	65 (18.2)	31 (14.6)	34 (23.4)	0.032	0/358
Length of ICU stay (days)	18 (1–170)	21 (6–170)	10 (1–79)	<0.001	0/358
ICU admission reason					0/358
Respiratory failure	81 (22.6)	52 (24.4)	29 (20.0)	0.014	
Cardiac nonsurgical	187 (52.2)	107 (50.2)	80 (55.2)		
Cardiac surgery	75 (20.9)	50 (23.5)	25 (17.2)		
Trauma	3 (0.8)	2 (0.9)	1 (0.7)		
Hypothermia	12 (3.4)	2 (0.9)	10 (6.9)		
ICU department					0/358
ICU 1	193 (53.9)	122 (57.3)	71 (49.0)	0.121	
ICU 2	165 (46.1)	91 (42.7)	74 (51.0)		
Mortality-related outcomes					0/358
Admission to death (days)	10 (1–88)	-	-		
ECMO initiation to death (days)	9 (1–87)	-	-		
Survived beyond ECMO support	277 (77.4)	-	-		
Survived beyond ICU	227 (63.4)	-	-		
Discharged alive	216 (60.3)	-	-		
Survived beyond one year	206 (57.5)	-	-		

* Data presented as mean ± standard deviation, median (minimum–maximum range) or number of patients (%). For clarity, mean is added if median of compared variables is same. Abbreviations: SAPS III, Simplified Acute Physiology Score III; SOFA, Sequential Organ Failure Assessment score; ICU, intensive care unit; ECMO, extracorporeal membrane oxygenation; CPR, cardiopulmonary resuscitation; ICU 1, General and surgical ICU; ICU 2, Traumatology ICU.

**Table 2:** ECMO-related characteristics and outcomes (*n* = 358, all-cause three-month mortality).

Clinical Characteristics *	All Patients (*n* = 358)	Survivors (*n* = 213)	Nonsurvivors (*n* = 145)	*p*-Value	Missing Data (*n*/Total)
ECMO support indications					0/358
Cardiogenic shock	258 (72.1)	153 (71.8)	105 (72.4)		
Respiratory failure	88 (24.6)	58 (27.2)	30 (20.7)	0.014	
Hypothermia	12 (3.4)	2 (0.9)	10 (6.9)		
Type of ECMO support					0/358
Venoarterial	284 (79.3)	165 (77.5)	119 (82.1)	0.163	
Venovenous	74 (20.7)	48 (22.5)	26 (17.9)		
ECMO-related clinical course					0/358
ECMO support duration (days)	6 (1–36)	6 (1–36)	6 (1–36)	0.315	
ECMO support duration < 7 days	238 (66.5)	147 (69.0)	91 (62.1)	0.218	
Time from admission to ECMO initiation (days)	0 (0–36)	0 (0–9)	0 (0–36)	0.449	
Day of ECMO support initiation					0/358
Week day	289 (80.7)	181 (85.0)	108 (74.5)	0.013	
Weekends	69 (19.3)	32 (15.0)	37 (25.5)		
Anticoagulation during ECMO support					1/358
UFH	278 (77.9)	176 (82.6)	102 (70.8)	0.015	
Argatroban	32 (9.0)	19 (8.9)	13 (9.0)		
Epoprostenol	1 (0.3)	0 (0.0)	1 (0.7)		
Argatroban and epoprostenol	5 (1.4)	2 (0.9)	3 (2.1)		
None	41 (11.5)	16 (7.5)	25 (17.4)		
Reason for ECMO support termination					0/358
Improvement (weaned)	252 (70.4)	195 (91.5)	57 (39.3)	<0.001	
Bridge to other assistance (heart transplant or VAD)	18 (5.0)	16 (7.5)	2 (1.4)		
Haemorrhage	7 (2.0)	2 (0.9)	5 (3.5)		
Death	81 (22.6)	-	81 (55.9)		

* Data presented as mean ± standard deviation, median (minimum–maximum range) or number of patients (%). Abbreviations: ECMO, extracorporeal membrane oxygenation; UFH, unfractionated heparin, VAD, ventricular assist device.

**Table 3:** Adverse events and blood products substitution during ECMO support (*n* = 358).

Complications *	All Patients (*n* = 358)	Survivors (*n* = 213)	Nonsurvivors (*n* = 145)	*p*-Value	Missing Data (*n*/Total)
Haemorrhage	160 (44.7)	79 (37.1)	81 (55.9)	<0.001	0/358
Major haemorrhage	96 (26.8)	43 (20.2)	53 (36.6)	0.001	0/358
Minor haemorrhage	64 (17.9)	36 (16.9)	28 (19.3)	0.559	0/358
Day of haemorrhage	1 (1–14)	1 (1–14)	1 (1–14)	0.589	0/358
Haemorrhage on the first ECMO support day	86 (24.0)	42 (19.7)	44 (30.3)	0.883	0/358
Haemorrhage within first three days	117 (32.7)	56 (26.3)	61 (42.1)	0.533	0/358
Coagulopathy	46 (12.8)	22 (10.3)	24 (16.6)	0.084	0/358
Thrombosis	82 (22.9)	48 (22.5)	34 (23.4)	0.840	0/358
Thrombosis venous	56 (15.6)	36 (16.9)	20 (13.8)	0.427	0/358
Thrombosis arterial	40 (11.2)	19 (8.9)	21 (14.5)	0.101	0/358
Sepsis	71 (19.8)	33 (15.5)	38 (26.2)	0.013	0/358
Substitution of blood products during ECMO support					26/358
Packed red blood cells (units)	6 (0–60)	5 (0–36)	8 (0–60)	<0.001	
Fresh-frozen plasma (units)	0 (0–92) 3.4 ± 8.6	0 (0–40) 2.6 ± 6.5	0 (0–92) 4.5 ± 10.8	0.022	
Platelets (units)	1 (0–30)	0 (0–30)	1 (0–22)	0.006	
Fibrinogen (g)	0 (0–26) 3.0 ± 4.9	0 (0–20) 2.2 ± 3.9	0 (0–26) 4.1 ± 5.9	0.003	
Antithrombin (IU)	0 (0–32,000)	0 (0–32,000)	0 (0–15,500)	0.478	
Prothrombin complex concentrate (IU)	0 (0–7200) 436 ± 1034.2	0 (0–3600) 272 ± 700.2	0 (0–7200) 677 ± 1353.3	0.005	
Factor XIII concentrate (IU)	0 (0–10,000)	0 (0–6250)	0 (0–10,000)	0.089	
Desmopressin (µg)	0 (0–30)	0 (0–30)	0 (0–30)	0.773	
Von Willebrand Factor (IU)	0 (0–5000) 104 ± 548.9	0 (0–5000) 76 ± 537.3	0 (0–4000) 145 ± 564.9	0.021	

* Data presented as mean ± standard deviation, median (minimum–maximum range) or number of patients (%). For clarity, mean and standard deviation added if median 0 and *p* < 0.05. Abbreviations: ECMO, extracorporeal membrane oxygenation; IU, international units.

**Table 4:** Identification of risk factors for death: multivariate analysis (*n* = 358).

Nondependent Variable	B-Coefficient	*p*-Value	HR	95% Confidence Interval	
				Lower	Upper
ECMO initiation on weekends	0.471	0.016	1.60	1.09	2.35
Resuscitation before ECMO	0.189	0.352	1.21	0.81	1.81
SAPS III score	0.037	<0.001	1.04	1.03	1.05
ECMO indication respiratory failure (reference category)					
Cardiogenic shock	0.410	0.066	1.51	0.97	2.33
Hypothermia	1.321	<0.001	3.75	1.79	7.87
Haemorrhage	0.551	0.001	1.74	1.24	2.43
Sepsis	0.180	0.376	1.20	0.80	1.78

Variables with increased hazard ratio for mortality: ECMO initiation on weekends, SAPS III score, hypothermia as indication for ECMO initiation and haemorrhage. Abbreviations: ECMO, extracorporeal membrane oxygenation; SAPS III, Simplified Acute Physiology Score III; ICU, intensive care unit; CI, confidence intervals; HR, hazard ratio. (Cases with missing data: 17/358).

## Data Availability

The datasets used and analysed during the current study are made available from the corresponding author on reasonable request.

## Supplementary Materials

The following supporting information can be downloaded at: https://www.mdpi.com/article/10.3390/jcm11051224/s1, Supplementary Figure S1. Comparison of patients based on the ICU admission reason and all-cause three-month mortality (n = 358); Supplementary Figure S2. Kaplan–Meier estimate of all-cause three-month mortality (n = 358, mean: 59.5, 95% CI: 55.5–63.5); Supplementary Table S1. Laboratory parameters within 24 h prior to ECMO initiation (n = 358); Supplementary Table S2. Subgroup analysis: ECMO initiation on working day or weekend; baseline demographic and clinical characteristics (n = 358); Supplementary Table S3. Selected demographic and clinical characteristics of patients according to the ECMO indication (n = 358); Supplementary Table S4. Venoarterial ECMO configuration: baseline demographic and clinical characteristics (n = 283, all-cause three-month mortality); Supplementary Table S5. Venovenous ECMO configuration: baseline demographic and clinical characteristics (n = 75, all-cause three-month mortality); Supplementary Table S6. Risk factors for mortality within three months from ECMO initiation: univariate Cox regression analyses (n = 358).
