# Supplementary material for: ECMO Predictors of Mortality: A 10-Year Referral Centre Experience
Source: J Clin Med. 2022 Feb 24;11(5):1224. doi: 10.3390/jcm11051224 (PMC8911127; doi:10.3390/jcm11051224)
Supplement: Supplementary file 1 [file jcm-11-01224-s001.zip › jcm-1597568-supplementary.pdf]

Journal of
Clinical Medicine

MDPI

Article

ECMO Predictors of Mortality: A 10-Year Referral Centre Experience

Benedikt Treml, Robert Breitkopf, Zoran Bukumirić, Mirjam Bachler, Johannes Boesch and Sasa Rajsic *

Supplementary material

Supplementary Figure S1. Comparison of patients based on the ICU admission reason and all-cause three-month mortality (*n* = 358).
Supplementary Figure S2. Kaplan-Meier estimate of all-cause three-month mortality (*n* = 358, Mean 59.5, 95% CI 55.5-63.5).
Supplementary Table S1. Laboratory parameters within 24h prior to ECMO initiation (*n* = 358)
Supplementary Table S2. Subgroup analysis: ECMO initiation on working day or weekend; baseline demographic and clinical characteristics (*n* = 358)
Supplementary Table S3. Selected demographic and clinical characteristics of patients according to the ECMO indication (*n* = 358)
Supplementary Table S4. Veno-arterial ECMO configuration: baseline demographic and clinical characteristics (*n* = 283, all-cause three-month mortality)
Supplementary Table S5. Veno-venous ECMO configuration: baseline demographic and clinical characteristics (*n* = 75, all-cause three-month mortality)
Supplementary Table S6. Risk factors for mortality within three months from ECMO initiation: univariate cox regression analyses (*n* = 358)

Supplementary Figure S1. Comparison of patients based on the ICU admission reason and all-cause three-month mortality (*n* = 358). Blue colour: survivors, *n* = 213; red colour: non-survivors, *n* = 145. Abbreviations: ICU, intensive care unit; CPR, cardiopulmonary resuscitation; CABG, Coronary artery bypass graft; STEMI, ST-elevation myocardial infarction; ARDS, Acute respiratory distress syndrome.

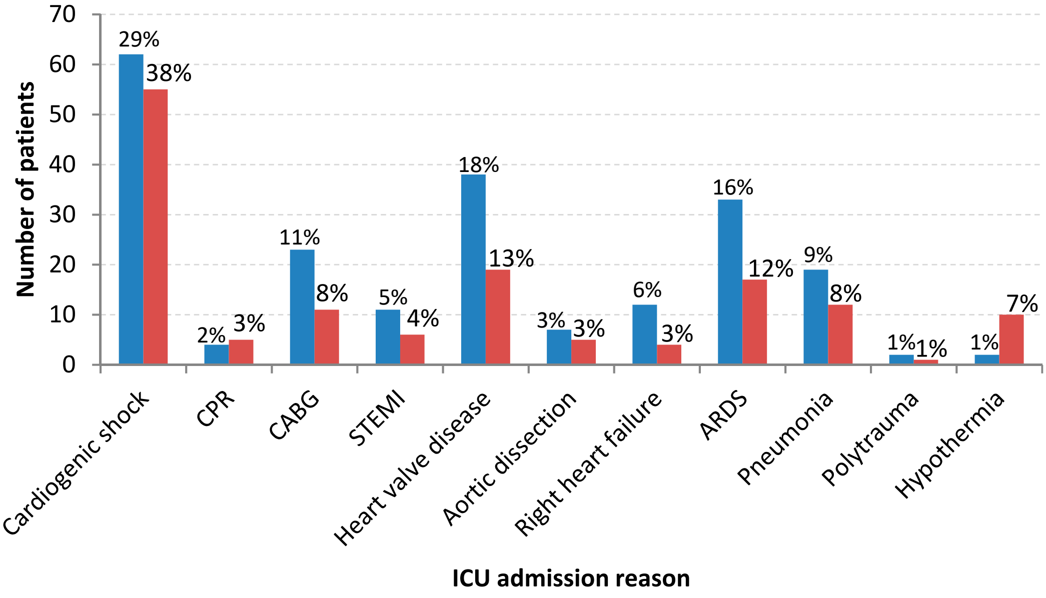

Supplementary Figure S2. Kaplan-Meier estimate of all-cause three-month mortality (n = 358, mean 59.5, 95% CI 55.5-63.5).

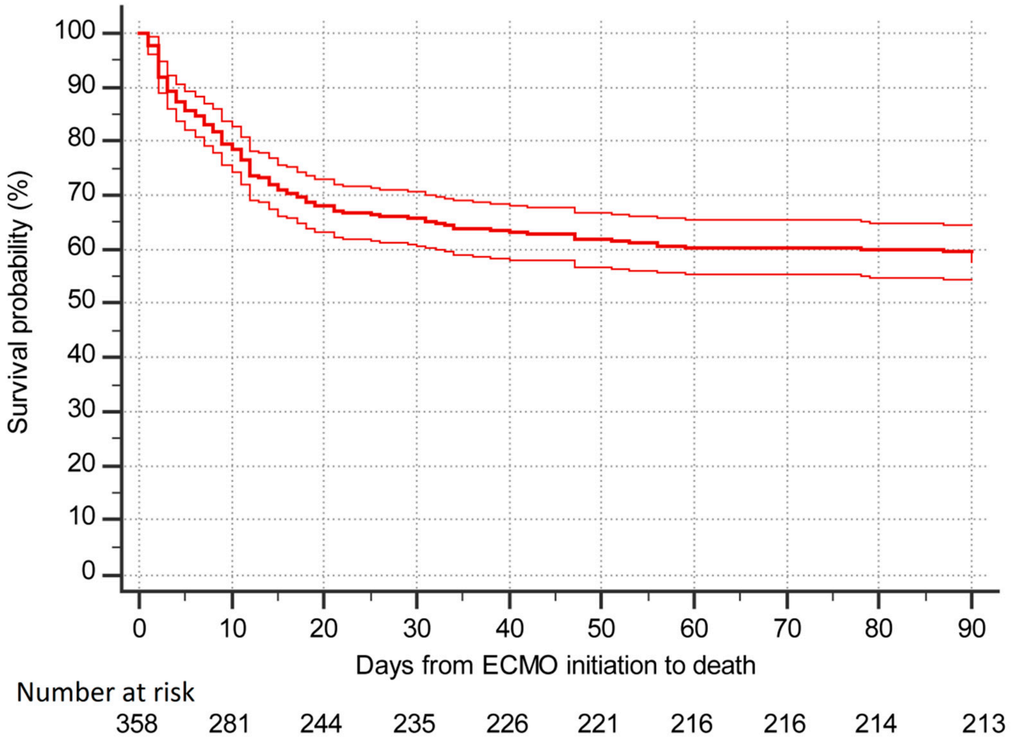

Supplementary Table S1. Laboratory parameters within 24h prior to ECMO initiation (n = 358)

**Laboratory parameters***	**All patients (n = 358)**	**Survivors (n = 213)**	**Non-survivors (n = 145)**	**p-value**	**Missing data (n/total)**
Haemoglobin (g/dL)	118.9 ±24.6	120.8 ±25.3	115.6 ±23.2	0.125	27/358
Red blood cells (T/L)	4.0 ±0.8	4.0 ± 0.8	3.9 ± 0.8	0.212	27/358
Haematocrit (%)	0.4 ±0.1	0.4 ±0.1	0.3 ±0.1	0.250	27/358
Leucocytes (g/L)	9.4 (2.2-51.5)	9.0 (2.2-39.4)	9.8 (3.7-51.5)	0.129	27/358
C-reactive protein (mg/L)	1.6 (0.1-50.8)	1.3 (0.1-48.2)	3.0 (0.1-50.8)	0.021	27/358
Procalcitonin (µg/L)	1.1 (0.1-125.9)	1.2 (0.1-125.9)	1.0 (0.1-46.2)	0.788	53/358
Platelets (g/L)	166 (18-464)	176 (29-464)	154 (18-423)	0.275	27/358
International normalized ratio	1.1 (0.9-6)	1.1 (0.9-6)	1.2 (0.9-6)	0.128	32/358
Activated partial thromboplastin time (s)	37 (24-201)	37 (24-201)	37 (24-201)	0.310	47/358
Prothrombin time (%)	80 (9-114)	81 (9-114)	75 (10-111)	0.228	47/358
Fibrinogen (mg/dL)	353 (57-1104)	336 (57-1104)	370 (69-1055)	0.141	31/358
Antithrombin (%)	79 (15-121)	81 (21-117)	78 (15-121)	0.872	145/358
Factor XIII (%)	66 (28-109)	69 (28-109)	55 (39-97)	0.211	311/358
InTEM Clotting Time (s)	183 (86-354)	201 (143-354)	176 (86-228)	0.028	316/358
InTEM Maximum Clot Firmness (mm)	56 (36-74)	56 (39-72)	61 (36-74)	0.740	316/358
FibTEM Maximum Clot Firmness (mm)	17 (5-34)	18 (5-30)	15 (7-34)	1.000	315/358

*Data presented as mean ± standard deviation or median (minimum – maximum range). Abbreviations: ECMO: extracorporeal membrane oxygenation.

Journal of
Clinical Medicine

MDPI

Supplementary Table S2. Subgroup analysis: ECMO initiation on working day or weekend; baseline demographic and clinical characteristics (*n* = 358)

Characteristics*	Working day (*n*=289)	Weekend (*n*=69)	p-value	Missing data (*n*/total)
Age (years)	59.4 ±15.7	55.7 ±16.6	0.083	0/358
Male sex	203 (70.2)	48 (69.6)	1.000	0/358
Body mass index (kg/m²)	27.3 ±5.5	26.8 ±4.1	0.389	10/358
SOFA score	12 (2-21)	12 (3-20)	0.760	0/358
SAPS III Score	65 (28-117)	70 (34-104)	0.036	1/358
CPR before ECMO initiation	49 (17.0)	16 (23.2)	0.228	0/358
ECMO duration (days)	6 (1-36)	6 (2-36)	0.565	0/358
ICU length of stay (days)	17 (1-170)	18 (2-98)	0.401	0/358
Admission reason				0/358
Respiratory disease	65 (22.5)	16 (23.2)		
Cardiac non-surgical	147 (50.9)	40 (58.0)		
Cardiac surgery	65 (22.5)	10 (14.5)	0.543	
Trauma	3 (1.0)	0 (0.0)		
Hypothermia	9 (3.1)	3 (4.3)		
Reason for ECMO support termination				0/358
Improvement	206 (71.3)	46 (66.7)		
Death	59 (20.4)	20 (29.0)	0.278	
Successful bridging	15 (5.2)	3 (4.3)		
Haemorrhage	9 (3.1)	0 (0.0)		
Complications				0/358
Haemorrhage	136 (47.1)	24 (34.8)	0.080	
Thrombosis	64 (22.1)	18 (26.1)	0.524	
Sepsis	56 (19.4)	15 (21.7)	0.737	
Mortality-related outcomes				0/358
Death on ECMO	61 (21.1)	20 (29.0)	0.199	
ICU-mortality	100 (34.6)	31 (44.9)	0.126	
3-months mortality	108 (37.4)	37 (53.6)	0.020	
1-year mortality	114 (39.4)	38 (55.1)	0.021	
Cause of death				0/358
Multiple organ failure	34 (31.5)	10 (27.0)		
Cardiac cause	39 (36.1)	10 (27.0)		
Respiratory failure	5 (4.6)	2 (5.4)	0.198	
Sepsis	12 (11.1)	2 (5.4)		
Brain death	15 (13.9)	9 (24.3)		
Other	3 (2.8)	4 (10.8)		

*Data presented as mean ± standard deviation, median (minimum – maximum range) or number of patients (%). Abbreviations: SAPS III, simplified acute physiology score III; SOFA, sequential organ failure assessment score; ICU, intensive care unit; ECMO, extracorporeal membrane oxygenation; CPR, cardiopulmonary resuscitation.

Supplementary Table S3. Selected demographic and clinical characteristics of patients according to the ECMO indication (*n* = 358)

Patient characteristics*	Cardiac shock (*n* = 258)	Respiratory failure (*n* = 88)	Hypothermia (*n* = 12)	p-value[1]	CS vs RF	CS vs HT	RF vs HT	Missing data (*n*/total)
Age (years)	62.8 ±13.7	48.6 ±16.4	42.3 ±16.0	<0.001	<0.001	<0.001	0.333	0/358
Male sex	174 (67.4)	70 (79.6)	7 (58.3)	0.067	-	-	-	0/358
Body mass index (kg/m²)	27.0 ±4.6	28.3 ±6.8	24.3 ±1.5	0.019	0.094	0.195	0.046	10/358
SOFA score	12 (2-20)	12 (3-21)	13 (10-17)	0.332	-	-	-	0/358
SAPS III Score	66 (28-104)	67 (31-117)	78 (58-89)	0.009	0.579	0.011	0.073	1/358
ECMO duration (days)	6 (1-17)	8 (1-36)	3 (1-25)	<0.001	<0.001	0.069	<0.001	0/358
ICU length of stay	6 (1-17)	20 (2-98)	3 (1-53)	<0.001	0.163	0.004	<0.001	0/358
CPR before ECMO initiation	53 (20.5)	7 (7.9)	5 (41.6)	0.003	0.006	0.063	0.003	0/358
Weekend	50 (19.4)	16 (18.0)	3 (25.0)	0.851	-	-	-	0/358
Complications and mortality-related outcomes								0/358
Death on ECMO	54 (20.9)	21 (23.6)	6 (50.0)	0.060	-	-	-	
ICU – mortality	95 (36.8)	26 (29.6)	10 (83.3)	0.001	0.296	0.002	0.024	
In-hospital mortality	103 (39.9)	29 (33.0)	10 (83.3)	0.004	0.256	0.001	0.005	
3-months mortality	105 (40.7)	30 (34.1)	10 (83.3)	0.005	0.312	0.005	0.003	
One-year mortality	111 (43.0)	31 (35.2)	10 (83.3)	0.006	0.212	0.007	0.003	
Bleeding event	121 (46.9)	35 (39.3)	4 (33.3)	0.369	-	-	-	
Major bleeding event	71 (27.5)	24 (27.0)	1 (8.3)	0.401	-	-	-	
Thrombosis	62 (24.0)	18 (20.2)	2 (16.7)	0.688	-	-	-	
Sepsis	33 (12.8)	37 (41.6)	1 (8.3)	<0.001	<0.001	1.000	0.048	
Cause of death								0/358
Multiple organ failure	30 (28.6)	13 (41.9)	1 (8.3)	<0.001	<0.001	0.001	0.034	
Cardiac decompensation	47 (44.8)	2 (6.5)	0 (0.0)					
Respiratory failure	2 (1.9)	5 (16.1)	0 (0.0)					
Sepsis	6 (5.7)	6 (19.4)	2 (16.7)					
Brain death	14 (13.3)	4 (12.9)	7 (58.3)					
Other	6 (5.7)	1 (3.2)	0 (0.0)					

*Data presented as mean ± standard deviation, median (minimum – maximum range) or number of patients (%). P-value[1]: cardiac shock versus respiratory failure versus hypothermia. Abbreviations: CS: cardiac shock; RF: respiratory failure, HT: hypothermia; SAPS III, simplified acute physiology score III; SOFA, sequential organ failure assessment score; ICU, intensive care unit; ECMO, extracorporeal membrane oxygenation; CPR, cardiopulmonary resuscitation.

Journal of
Clinical Medicine

Supplementary Table S4. Veno-arterial ECMO configuration: baseline demographic and clinical characteristics (*n* = 283, all-cause three-month mortality)

Patient characteristics*	All patients (*n* = 283)	Survivors (*n* = 164)	Non-survivors (*n* = 119)	p-value	Missing data (*n*/total)
Age (years)	61.4 ±14.8	61.0 ±14.5	61.9 ±15.2	0.617	0/283
Male sex	190 (67.1)	112 (68.3)	78 (65.5)	0.627	0/283
SAPS III score	66 (28-104)	60 (28-97)	73 (41-104)	<0.001	1/283
SOFA score	12 (2-20)	11 (3-19)	12 (2-20)	0.015	0/283
CPR before ECMO initiation	59 (20.8)	25 (15.2)	34 (28.6)	0.006	0/283
Length of ICU stay (days)	16 (1-170)	21 (6-170)	9 (1-79)	<0.001	0/283
ICU admission reason					0/283
Respiratory failure	13 (4.6)	6 (3.7)	7 (5.9)		
Cardiac non-surgical	182 (64.3)	105 (64.0)	77 (64.7)		
Cardiac surgery	75 (26.5)	50 (30.5)	25 (21.0)	0.016	
Trauma	1 (0.4)	1 (0.6)	0 (0.0)		
Hypothermia	12 (4.2)	2 (1.2)	10 (8.4)		
Mortality related outcomes					0/283
ECMO initiation to death (days)	9 (1-79)	-	-		
Death during ICU	109 (38.5)				
Discharged alive	166 (58.7)	-	-		
ECMO support indications					0/283
Cardiogenic shock	258 (91.2)	153 (93.3)	105 (88.2)		
Respiratory failure	13 (4.6)	9 (5.5)	4 (3.4)	0.010	
Hypothermia	12 (4.2)	2 (1.2)	10 (8.4)		
ECMO related clinical course					0/283
ECMO support duration (days)	6 (1-25)	6 (1-25)	5 (1-25)	0.284	
Time from admission to ECMO initiation (days)	0 (0-20)	0 (0-9)	0 (0-20)	0.734	
Day of ECMO support initiation					0/283
Week day	226 (79.9)	140 (85.4)	86 (72.3)	0.007	
Weekend	57 (20.1)	24 (14.6)	33 (27.7)		
Anticoagulation during ECMO support					1/283
UFH	217 (76.7)	135 (82.3)	82 (68.9)		
Argatroban	26 (9.2)	14 (8.5)	12 (10.1)	0.025	
Argatroban and epoprostenol	4 (1.4)	1 (0.6)	3 (2.5)		
None	36 (12.7)	14 (8.5)	22 (18.5)		
Reason for ECMO support termination					0/283
Improvement (weaned)	196 (69.3)	147 (89.6)	49 (41.2)		
Bridge to other assistance (heart transplant or VAD)	18 (6.4)	16 (9.8)	2 (1.7)	<0,001	
Haemorrhage	6 (2.1)	1 (0.6)	5 (4.2)		
Death	63 (22.3)	-	63 (52.9)		
Complications					0/283
Haemorrhage	130 (54.1)	62 (37.8)	68 (57.1)	0.001	
Major haemorrhage	75 (26.5)	32 (19.5)	43 (36.1)	0.002	
Minor haemorrhage	55 (19.4)	30 (18.3)	25 (21.0)	0.569	
Day of haemorrhage	1 (1-18)	1 (1-14)	1 (1-18)	0.865	
Coagulopathy	32 (11.3)	14 (8.5)	18 (15.1)	0.084	
Thrombosis	67 (23.7)	39 (23.8)	28 (23.5)	0.961	
Thrombosis venous	42 (14.8)	27 (16.5)	15 (12.6)	0.367	

Thrombosis arterial	36 (12.7)	18 (11.0)	18 (15.1)	0.301	
Sepsis	36 (12.7)	15 (9.1)	21 (17.6)	0.034	
Substitution of blood products during ECMO support					17/283
Packed red blood cells (units)	6 (0-60)	6 (0-36)	8 (0-60)	0.017	
Fresh-frozen plasma (units)	0 (0-92)<br>4.1 ±9.4	0 (0-40)<br>3.2 ±7.3	0 (0-92)<br>5.3 ±11.7	0.037	
Platelets (units)	1 (0-22)	1 (0-10)	1 (0-22)	0.064	
Fibrinogen (g)	0 (0-26)<br>3.1 ±4.8	0 (0-16)<br>2.5 ±3.9	0 (0-26)<br>4.1 ±5.5	0.020	
Antithrombin (IU)	0 (0-17266)	0 (0-17266)	0 (0-15500)	0.679	
Prothrombin complex concentrate (IU)	0 (0-7200)<br>493 ±1095	0 (0-3600)<br>308 ±745	0 (0-7200)<br>747 ±1410	0.011	
Factor XIII concentrate (IU)	0 (0-10000)	0 (0-6250)	0 (0-10000)	0.264	
Desmopressin (µg)	0 (0-30)	0 (0-30)	0 (0-30)	0.788	
Von Willebrand Factor (IU)	0 (0-5000)<br>88 ±501	0 (0-5000)<br>43 ±420	0 (0-4000)<br>151 ±591	0.007	

*Data presented as mean ± standard deviation, median (minimum – maximum range) or number of patients (%). For clarity, mean and standard deviation added if median is 0 and p<0.05. Abbreviations: ECMO, extracorporeal membrane oxygenation; SAPS III, simplified acute physiology score III; SOFA, sequential organ failure assessment score; ICU, intensive care unit; ECMO, extracorporeal membrane oxygenation; CPR, cardiopulmonary resuscitation; UFH, Unfractionated Heparin, VAD, ventricular assist device, IU, international units.

Supplementary Table S5. Veno-venous ECMO configuration: baseline demographic and clinical characteristics (*n* = 75, all-cause three-month mortality)

Patient characteristics*	All patients (*n* = 75)	Survivors (*n* = 49)	Non-survivors (*n* = 26)	p-value	Missing data (*n*/total)
Age (years)	48.2 ±16.0	46.1 ±15.5	52.1 ±16.6	0.135	0/75
Male sex	61 (81.3)	39 (79.6)	22 (84.6)	0.759	0/75
SAPS III score	67 (31-117)	66 (35-112)	73 (31-117)	0.037	0/75
SOFA score	12 (3-21)	12 (3-21)	13 (3-21)	0.053	0/75
CPR before ECMO initiation	6 (8.0)	6 (12.2)	0 (0.0)	0.087	0/75
Length of ICU stay (days)	20 (9-98)	21 (10-98)	18 (2-49)	0.032	0/75
ICU admission reason					0/75
Respiratory failure	68 (90.7)	46 (93.9)	22 (84.6)	0.413	
Cardiac non-surgical	5 (6.7)	2 (4.1)	3 (11.5)		
Trauma	2 (2.7)	1 (2.0)	1 (3.8)		
Mortality related outcomes					0/75
Admission to death (days)	18 (2-88)	-	-		
ECMO initiation to death (days)	14 (1-87)	-	-		
Death during ICU	22 (29.3)	-	22 (84.6)		
Survived beyond ECMO support	57 (76.0)	-	-		
Discharged alive	50 (66.7)	-	-		
ECMO related clinical course					0/75
ECMO support duration (days)	9 (1-36)	8 (1-36)	11 (1-36)	0.381	
Time from admission to ECMO initiation (days)	0 (0-36)<br>1.5 ±4.9	0 (0-9)<br>0.7 ±1.9	0 (0-36)<br>3.0 ±7.7	0.029	
Day of ECMO support initiation					0/75
Week day	63 (84.0)	41 (83.7)	22 (84.6)	0.007	
Weekend	12 (16.0)	8 (16.3)	4 (15.4)		
Anticoagulation during ECMO support					0/75
UFH	61 (82.4)	41 (83.7)	20 (80.0)	0.324	

Argatroban	6 (8.1)	5 (10.2)	1 (4.0)		
Epoprostenol	1 (1.4)	0 (0.0)	1 (4.0)		
Argatroban and epoprostenol	1 (1.4)	1 (2.0)	0 (0.0)		
None	5 (6.8)	2 (4.1)	3 (12.0)		
Reason for ECMO support termination					0/75
Improvement (weaned)	56 (74.7)	48 (98.0)	8 (30.8)		
Haemorrhage	1 (1.3)	1 (2.0)	0 (0.0)	<0.001	
Death	18 (24.0)	-	18 (69.2)		
Complications					0/75
Haemorrhage	30 (40.0)	17 (34.7)	13 (50.0)	0.198	
Major haemorrhage	21 (28.0)	11 (22.4)	10 (38.5)	0.142	
Minor haemorrhage	9 (12.0)	6 (12.2)	3 (11.5)	0.929	
Day of haemorrhage	4 (1-18)	5 (1-18)	4 (1-11)	0.536	
Coagulopathy	14 (18.7)	8 (16.3)	6 (23.1)	0.540	
Thrombosis	15 (20.0)	9 (18.4)	6 (23.1)	0.627	
Thrombosis venous	14 (18.7)	9 (18.4)	5 (19.2)	0.927	
Thrombosis arterial	4 (5.3)	1 (2.0)	3 (11.5)	0.117	
Sepsis	35 (46.7)	18 (36.7)	17 (65.4)	0.018	
Substitution of blood products during ECMO support					9/75
Packed red blood cells (units)	4 (0-33)	2 (0-31)	8 (0-33)	0.001	
Fresh-frozen plasma (units)	0 (0-10)	0 (0-6)	0 (0-10)	0.981	
Platelets (units)	0 (0-30)	0 (0-30)	1 (0-12)	0.015	
	2.1 ±4.7	1.9 ±5.2	2.4 ±3.6		
Fibrinogen (g)	0 (0-22)	0 (0-20)	0 (0-22)	0.135	
Antithrombin (IU)	0 (0-32000)	0 (0-32000)	0 (0-9500)	0.453	
Prothrombin complex concentrate (IU)	0 (0-4500)	0 (0-2000)	0 (0-4500)	0.334	
Factor XIII concentrate (IU)	0 (0-6750)	0 (0-5000)	0 (0-6750)	0.104	
Desmopressin (μg)	0 (0-30)	0 (0-30)	0 (0-30)	0.961	
Von Willebrand Factor (IU)	0 (0-4000)	0 (0-4000)	0 (0-2000)	0.837	

*Data presented as mean ± standard deviation, median (minimum – maximum range) or number of patients (%). For clarity, mean and standard deviation added if median is 0 and p<0.05. Abbreviations: ECMO, extracorporeal membrane oxygenation; SAPS III, simplified acute physiology score III; SOFA, sequential organ failure assessment score; ICU, intensive care unit; ECMO, extracorporeal membrane oxygenation; CPR, cardiopulmonary resuscitation; UFH, Unfractionated Heparin, VAD, ventricular assist device, IU, international units.

Supplementary Table S6. Risk factors for mortality within three months from ECMO initiation: univariate cox regression analyses (*n* = 358)

Nondependent variable*	B-coefficient	P-value	HR	95% confidence interval	
				lower	upper
Age (years)	0.009	0.121	1.01	1.00	1.02
Sex (male/female)	0.048	0.789	1.05	0.74	1.49
Height (cm)	-1.230	0.139	0.29	0.06	1.49
Weight (kg)	-0.002	0.739	1.00	1.00	1.01
Body mass index (kg/m2)	0.012	0.464	1.01	0.98	1.04
SAPS III score	0.039	<0.001	1.04	1.03	1.05
SOFA Score	0.069	0.002	1.07	1.03	1.12
Reanimation before ECMO	0.471	0.016	1.60	1.09	2.35
ICU Department	0.254	0.126	1.29	0.93	1.79
Observation period (year)	-0.051	0.101	0.95	0.90	1.01
ICU length of stay	-0.081	<0.001	0.92	0.90	0.94
Type of ECMO	-0.059	0.737	0.94	0.67	1.33

ECMO duration	-0.013	0.457	0.99	0.95	1.02
ECMO initiation on weekend	0.450	0.018	1.57	1.08	2.28
Admission reason (reference category: respiratory disease)					
Cardiac non-surgical	0.257	0.236	1.29	0.85	1.98
Cardiac surgery	-0.009	0.974	0.99	0.58	1.69
Trauma	0.146	0.886	1.16	0.16	8.50
Hypothermia	1.518	<0.001	4.57	2.22	9.40
ECMO Indication (reference category: respiratory failure)					
Cardiogenic shock	0.230	0.260	1.26	0.84	1.88
Hypothermia	1.521	<0.001	4.58	2.17	9.65
Thrombosis	-0.024	0.901	0.98	0.67	1.43
Coagulopathy	0.340	0.128	1.41	0.91	2.18
Haemorrhage	0.574	0.001	1.78	1.28	2.46
Sepsis	0.361	0.056	1.44	0.99	2.08
Anticoagulation medication	-0.258	0.127	0.77	0.56	1.08
C-reactive protein (mg/dL)	0.014	0.136	1.01	0.99	1.03
Procalcitonin (µg/L)	-0.014	0.330	0.99	0.96	1.01

Variables with increased hazard ratio for mortality: ECMO initiation on weekend, admission due to hypothermia, reanimation before ECMO initiation, SAPS III and SOFA score, hypothermia as indication for ECMO support, haemorrhage, sepsis and ICU length of stay. Abbreviations: CI, confidence Intervals; HR, hazard ratio; SAPS III, simplified acute physiology score III; SOFA, sequential organ failure assessment score; ICU, intensive care unit; ECMO, extracorporeal membrane oxygenation.
